# Garcinia cambogia—A Supplement-Related Liver Injury

**DOI:** 10.7759/cureus.22225

**Published:** 2022-02-15

**Authors:** Loreto L Calaquian, Irene Yau

**Affiliations:** 1 Internal Medicine, Brian D. Allgood Army Community Hospital, Seoul, KOR; 2 Flight Medicine, Brian D. Allgood Army Community Hospital, Pyeongtaek, KOR

**Keywords:** drug-induced liver injury, garcinia cambogia, acute liver injury, supplement-related liver injury, liver damage

## Abstract

Drug-induced liver injury, and specifically supplement-related liver injury, accounts for an increasing proportion of acute liver injury cases. We present the case of acute liver injury due to a *Garcinia cambogia*-containing weight-loss supplement not previously associated with liver injury. Identifying supplements as a potential cause is a key to managing acute liver injury of uncertain etiology.

## Introduction

Drug-induced liver injury (DILI) is a frequently encountered etiology of acute liver injury. A specific subset of DILI, supplement-related injury, represents an increasingly large proportion of liver injury cases, likely reflecting the increased use of supplements by the general population [1]. With countless formulations being advertised annually to a progressively receptive patient population, identifying potentially harmful supplements and ingredients is more important than ever to guide the care of patients presenting with significant liver injury. We present the case of supplement-related liver injury in a patient who admitted to the long-term use of a weight-loss supplement not previously associated with liver injury.

## Case presentation

The patient is a 45-year-old woman who initially presented to an outside emergency care facility for progressive-onset jaundice. Over the prior week, she had noticed progressive yellowing of her skin and sclera associated with fatigue, malaise, and anorexia. She had otherwise been in her baseline state of health. Outside facility labs noted marked elevation in liver function test enzymes, notably with aspartate aminotransferase (AST) of 1491 U/L and alanine aminotransferase (ALT) of 894 U/L (Table 1).

**Table 1:** Laboratory Data AST: aspartate aminotransferase; ALT: alanine aminotransferase; INR: international normalized ratio

Variable	Reference Range	Outside ER labs	On Admission	Hospital Day 2	Hospital Day 23
Creatinine (mg/dL)	0.52-1.04		0.55	0.48	0.58
Total bilirubin (mg/dL)	0.2-1.3	13.7	13.6	14.4	20.7
Direct bilirubin (mg/dL)	0.00-0.40	11.4	11.1	11.7	19.1
INR	0.87-1.13		1.69	2.03	1.69
Albumin (g/dL)	3.5-5.0		3.1	2.6	2.7
AST (U/L)	14-36	1491	1332	1082	101
ALT (U/L)	4-35	894	>750	674	60
Platelet count (× 10^3^/µL)	163-356		202	156	262

The patient had no recent history of alcohol consumption or history of intravenous drug use. She denied any personal or family history of liver disease, hypercoagulable disorders, or malignancy. Baseline liver function test enzymes (AST, ALT, total bilirubin) had all previously been within normal limits on screening labs 6 years prior to presentation. She denied taking any prescription or over-the-counter medications except for a weight-loss supplement, Seryburn Day Triple (SERY BOX) that she had been taking daily for the past 3 months. On physical examination, she was notably jaundiced but had no evidence of encephalopathy, abdominal distension, asterixis, or stigmata of chronic liver disease. Vital signs were all within normal limits. Repeat labs at our institution confirmed the elevation in liver enzymes with a model for end-stage liver disease (MELD) score based on her labs of 22 (Table 1). Viral serologies demonstrated no evidence for acute viral hepatitis (Table 2).

**Table 2:** Viral Serologies

Variable	On Admission (Reference range)
Hepatitis A virus Ab IgM	Negative
Hepatitis B virus surface Ag	Negative
Hepatitis B virus surface Ab	Positive
Hepatitis B virus core Ab	Positive
Hepatitis B virus core Ab IgM	Negative
Hepatitis C virus Ab	Negative
Epstein-Barr virus capsid Ab IgM	Undetectable (0.0-35.9)
Epstein-Barr virus capsid Ab IgG (U/mL)	66 (0.0-17.9)
Epstein-Barr virus nuclear Ab IgG (U/mL)	34.7 (0.0-17.9)

**Figure 1:**
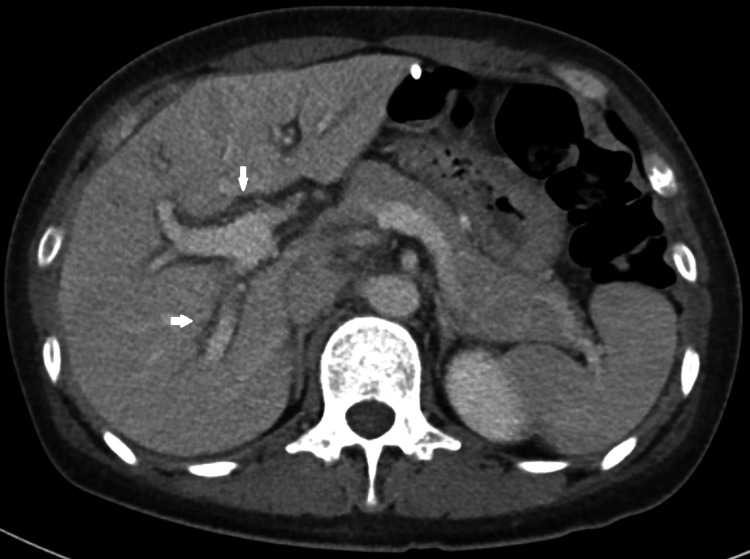
CT Abdomen/Pelvis with IV contrast (Axial view) A CT scan of the abdomen and pelvis obtained on admission showing mild hepatomegaly, diffuse irregularity of the hepatic contour, and periportal edema (white arrows).

Acetaminophen levels were undetectable, autoimmune markers were negative, and HIV antibody was negative. CT imaging of the abdomen obtained on admission found mild hepatomegaly, diffuse irregularity of the hepatic contour, and periportal edema without evidence of intra- or extrahepatic biliary ductal dilatation (Figure 1). A liver biopsy performed at an outside transplant center demonstrated submassive hepatic necrosis with marked ductal proliferation. The patient’s weight-loss supplement was immediately discontinued and she was admitted for observation. While the patient remained clinically stable, daily labs continued to demonstrate a persistent, worsening coagulopathy (international normalized ratio (INR 2.03)), and so she was transferred to an outside transplant center. The patient was treated with ursodeoxycholic acid, carnitine orotate complex, and milk thistle extract. The patient showed no evidence of further clinical decompensation or worsening liver injury, so she opted to continue conservative management and watchful waiting. During the remainder of her admission, liver enzymes and INR continued to downtrend to a normal range, and by the time of discharge nearly two weeks later her AST and ALT had dropped by over 50% (Table 1).

## Discussion

DILI accounts for over half of the cases of acute liver injury in the United States [2]. Because it is a diagnosis of exclusion, the actual proportion of DILI in the reported cases of liver injury is difficult to determine [3]. As in our case, patients who present with acute episodes of DILI often have non-specific constitutional symptoms and vague gastrointestinal complaints only suggestive of hepatobiliary illness. Furthermore, widespread underreporting of supplement usage compounds the difficulty linking supplement usage to the onset of liver injury. This is despite the increase in acceptance and popularity of supplement usage as a means for patients to enhance their health. In populations served by military treatment facilities, the percentage of patients regularly using supplements is reported to be as high as 60%. Despite this, only 36.6% of respondents from this population reported their dietary supplement use to their physician [4]. This emphasizes the importance of a thorough history and careful medication reconciliation with a detailed timeline of when medications, including supplements, were taken in relation to symptom onset in establishing a timely, accurate diagnosis [1].

The major active ingredient of Seryburn Day Triple is *Garcinia cambogia*, a fruit-bearing plant native to South and Southeast Asia. In these regions, Garcinia is typically used as cuisine flavoring, as a preservative agent for foods, and as a traditional medicine for ailments in both humans and cattle [5]. More recently, it has been studied and advertised as a weight-loss dietary supplement. One of the plant’s active metabolites is hydroxycitric acid (HCA), which is implicated in the reduction of fatty acid synthesis, reduced lipogenesis, increased fat loss with aerobic exercise, and appetite suppression. Evidence for these benefits has been relatively limited, but a potential association with liver injury due to high levels of HCA is of increasing concern [6-8].

In all cases of liver injury, the differential diagnosis must remain broad, and a thorough evaluation must rule out concomitant toxins and infections. In our patient, the positive hepatitis B virus core antibody in the absence of hepatitis B surface antigen is consistent with immunity due to prior infection of hepatitis B virus, which is endemic in South Korea (the patient’s country of origin), rather than acute viral hepatitis [9]. Epstein-Barr virus capsid and nuclear IgG antibody positivity in the absence of IgM positivity or clinical symptoms of infectious mononucleosis similarly represents prior infection rather than acute infection. A particular concern in all cases of supplement-related liver injury is the reality of minimal regulatory oversight of supplement production, and the possibility that subsequently identified adverse side effects and outcomes may be a result of unlabeled ingredients or contaminants as a result of the production process [10].

## Conclusions

In this case, our patient developed acute liver injury while taking a *Garcinia cambogia* supplement and demonstrated rapid clinical improvement following discontinuation of the supplement. The mechanism by which Garcinia causes hepatocellular damage is still unclear and has yet to be definitively elucidated. Garcinia itself is an uncommonly identified cause of liver injury, and the supplement Seryburn Day Triple has not been previously implicated in cases of DILI. Despite the increasingly widespread use of supplements, medical knowledge and methodical research remain limited, a significant issue when effective diagnosis and management relies on rapid identification of possible etiologies of disease. As ever in medicine, this shortcoming only serves to underscore the importance of reporting and documenting novel findings, including evidence for potential adverse effects, to further augment our collective medical knowledge and help guide clinical decision-making and patient management.
